# Dual-Drug Chitosan Hydrogel Loaded with Metformin and Cefazolin for Antibacterial, Anti-Inflammatory and Antioxidant Treatment of Drug-Resistant Bacteria-Infected Skin Wounds

**DOI:** 10.3390/gels12100876

**Published:** 2026-09-28

**Authors:** Yilin Zhang, Jie Cao, Yong Yan, Zhaoyang Li, Haiqiang Ren, Chenxiong Xu, Chengfang Qiao, Wei Zhou

**Affiliations:** 1 College of Modern Agriculture and Bioengineering, Shangluo University, Shangluo 726000, China; yilin_190@126.com (Y.Z.);; 2 College of Chemical Engineering and Modern Materials, Shangluo University, Shangluo 726000, China; 3 Shaanxi Xiangju Pharmaceutical Group Company Limited, Shangluo 726000, China

**Keywords:** chitosan hydrogel, metformin, cefazolin, antibiotic synergist, wound healing

## Abstract

Bacterial-infected skin wounds, especially those caused by drug-resistant pathogens accompanied by persistent inflammation, remain a major clinical therapeutic challenge. Rising antimicrobial resistance compromises the therapeutic effect of conventional single-agent antibiotics, creating an urgent demand for novel wound therapeutics that integrate bactericidal potency, immunomodulation and tissue regeneration capacity. In this work, an injectable multifunctional chitosan-based hydrogel (C@MC) was developed, which simultaneously incorporates metformin hydrochloride (as a potential antibiotic adjuvant) and cefazolin sodium (as a β-lactam class antibacterial drug). The optimized C@MC possesses interconnected three-dimensional pores with an initial storage modulus of ~0.83 kPa (C@MC_360_), featuring desirable mechanical strength, injectability, tissue adhesion and dual pH/glucose-responsive sustained drug release. In vitro evaluations revealed the dose-dependent bactericidal activity of C@MC against *Staphylococcus aureus* (*S. aureus*), *Escherichia coli* (*E. coli*) and New Delhi metallo-β-lactamase-1-producing *E. coli* (NDM-1 *E. coli*, resistant bacteria). The highest drug-laden group nearly eliminated all wound bacteria in vitro. It scavenged reactive oxygen species (ROS) to lower cellular MDA levels and suppress pro-inflammatory TNF-α and IL-6 in LPS-stimulated RAW 264.7 macrophages. In full-thickness mouse wounds infected with NDM-1 *E. coli*, C@MC_360_ achieved nearly complete wound closure, with only a 4.2% residual wound area on day 14, accompanied by suppressed inflammatory infiltration and accelerated wound regeneration. This dual-drug hydrogel integrates antibiotic potentiation, anti-inflammation and antioxidation, providing an intervention regimen for skin wounds infected with drug-resistant bacteria.

## 1. Introduction

The skin, the largest organ in the human body, serves as a crucial physical and chemical barrier against external pathogenic microorganisms. It also undertakes essential physiological functions, including temperature regulation and maintaining systemic homeostasis [1]. When trauma, burns or diabetes-related chronic lesions destroy the skin barrier, the subcutaneous tissue is easily colonized by microbes, which can trigger severe wound infection, systemic sepsis and even death [2]. Bacterial-infected skin wounds inflict a heavy disease burden worldwide, and drug-resistant bacterial infection further worsens wound prognosis [3].

β-lactam antibiotics remain the first-line therapeutic agents for cutaneous bacterial infection, accounting for approximately 65% of the global antibacterial market [4,5]. However, the widespread and unregulated misuse of antibiotics has triggered a global antimicrobial resistance (AMR) crisis. In 2019, roughly 1.27 million fatalities were directly attributed to infections triggered by drug-resistant bacteria [6]. Among resistant strains, New Delhi metallo-β-lactamase-1 (NDM-1)-producing resistant Enterobacteriaceae pose a major clinical challenge. NDM-1 can hydrolyze almost all β-lactam antibiotics, covering carbapenems known as the clinical “last line of defense” against infections [7,8]. The primary strategy for combating AMR mainly relies on developing new antibiotic scaffolds. Nevertheless, the rapid occurrence and dissemination of resistant strains is vastly outpacing the research and approval of new antibiotics, and there has been a lack of new antibiotic classes over the past three decades [9]. For this reason, it is imperative to explore alternative strategies that enhance the bactericidal efficacy of existing antibiotics against NDM-1-expressing resistant pathogens.

Hydrogels, as three-dimensional crosslinked polymer networks, have turned out to be promising biomaterials for wound dressing applications owing to their unique structural and functional advantages [10]. The high water retention capacity of hydrogels maintains a moist microenvironment favorable for skin regeneration. Their interconnected porous structure enables gas exchange and absorbs excess wound exudates, while their intrinsic flexibility allows for conformal attachment to irregular wound surfaces. Furthermore, gel matrices can physically encapsulate bioactive compounds to achieve sustained and tunable drug release [11]. In recent years, functional wound hydrogels have evolved beyond passive wound coverage to include stimuli-responsive drug release, chemodynamic/photothermal antibacterial activity, reactive oxygen species (ROS) scavenging, and macrophage immunomodulation within a single gel system [12,13,14]. Meanwhile, staged-release hydrogels that sequentially deliver antibacterial and anti-inflammatory payloads have also been developed to match the multistep repair cascade of damaged skin [15]. However, many composite hydrogels are limited by their rigid mechanical properties, low fluidity and poor injectability for irregular wounds [16]. Based on this background, chitosan (CS) was selected as the gel matrix, considering its good biocompatibility, biodegradability, intrinsic hemostatic activity, and abundant amino/hydroxyl functional groups that support physical crosslinking and high drug encapsulation efficiency [17,18,19].

Although extensive progress has been made in antibacterial hydrogel fabrication, few gel formulations are suitable to treat wounds infected by NDM-1-producing resistant bacteria. Commercial antibiotic-loaded dressings mostly contain a single drug, such as gentamicin (Collamycin^®^) [20] and fluoroquinolones (Otiprio^®^) [21], while clinically available β-lactam hydrogels are scarce and rarely deployed for wound care. Once exposed to NDM-1 strains, β-lactams are rapidly hydrolyzed by NDM-1 metalloenzymes, causing poor sterilization and persistent inflammation [22]. Moreover, NDM-1 infection also elevates intracellular ROS and pro-inflammatory TNF-α/IL-6, forming a vicious oxidative–inflammatory cycle that hinders granulation tissue growth. To address the AMR crisis, repurposing marketed drugs as antibiotic resistance breakers has become a viable alternative against multidrug-resistant bacterial infections [23].

Metformin hydrochloride (Met), a first-line oral hypoglycemic drug for type 2 diabetes mellitus, has attracted widespread attention due to its multiple pharmacological activities beyond hypoglycemic regulation [24,25]. Met can block the NF-κB signaling cascade to reduce pro-inflammatory cytokine release and alleviate AMPK pathways to upregulate endogenous antioxidant enzymes, thereby alleviating oxidative damage in lesional tissue [26,27,28]. More importantly, recent evidence validates Met as an effective antibiotic synergist capable of reversing multidrug resistance and recovering the bactericidal potency of β-lactam antibiotics [29]. Its dual anti-inflammatory and resistance-sensitizing properties make Met an underexplored yet highly promising bioactive additive for advanced anti-resistance wound hydrogels.

Herein, we aim to construct an injectable CS-based dual-drug hydrogel co-loaded with Met and cefazolin sodium (CFZ, a β-lactam class antibiotic). Met is introduced as a potential antibiotic adjuvant intended to restore and strengthen the antibacterial activity of CFZ against NDM-1-producing resistant bacteria. We systematically characterize the physicochemical properties; stimuli-responsive drug release; and antibacterial, antioxidant and immunomodulatory behaviors of composite hydrogels via a series of in vitro assays. Furthermore, a New Delhi metallo-β-lactamase-1-producing *Escherichia coli* (NDM-1 *E. coli*)-infected full-thickness mouse wound model is adopted to validate the wound repair efficacy of the optimized hydrogel. This work is expected to provide an alternative therapeutic strategy for skin wounds infected with drug-resistant bacteria.

## 2. Results and Discussion

### 2.1. Macroscopic Characteristics of C@MC

The preparation strategy was adapted from a reported reference [30], with partial optimizations implemented for our hydrogel system. Met and CFZ were mixed with preprepared CS solution, and continuously stirred for 12 h to promote sufficient supramolecular interactions between the drug molecules and CS chains. The mixture was then statically incubated for 6 h to generate a free-standing, transparent drug-laden composite hydrogel. Figure 1A presents the macroscopic morphology of C@MC_360_. After resting for 6 h, the hydrogel turned from clear to pale yellow. The initial mixture was a sol, whereas the final product formed a rigid gel without flowing upon inversion. The sol-to-gel transition relies on physical supramolecular crosslinking. In a dilute acetic acid medium, CS amino groups are protonated into −NH_3_^+^, while abundant hydroxyl groups are retained [31]. The polar moieties, including guanidine groups of Met and amide/carboxylate groups of CFZ, generate extensive intermolecular hydrogen bonds with the hydroxyl and protonated amino sites on the CS chains. Electrostatic attraction also occurs between the positively charged −NH_3_^+^ of CS and negatively charged carboxylate groups from CFZ. Cumulative non-covalent interactions raise the crosslinking density during the 6 h static incubation and trigger sol–gel conversion to construct a stable three-dimensional network.

### 2.2. FT-IR and UV-Vis Spectral Analysis of Hydrogels

Fourier transform infrared (FT-IR) spectroscopy was carried out to confirm hydrogel synthesis. Figure 1B displays the spectra of pure CS, C@gel, Met, CFZ, C@M_100_ and dual-drug hydrogels (C@MC_90_, C@MC_180_, and C@MC_360_).

For pure CS, the broad absorption peak centered at 3353.2 cm^−1^ corresponds to the overlapping stretching vibrations of O-H and N-H bonds, originating from the abundant hydroxyl and amino groups distributed along the CS chains. The blank C@gel shows a distinct C=O stretching signal at 1650.1 cm^−1^, which supports the formation of physically crosslinked networks. Met shows characteristic signals at 1048.5 cm^−1^ (C-O-C stretch) and 1507.0 cm^−1^ (N-H bending), while CFZ produces a broad absorption at 3301.4 cm^−1^ from overlapping hydroxyl and amino vibrations.

Notably, no new absorption peaks indicative of covalent bond formation were observed in the spectra of drug-loaded hydrogels. The variations in peak intensity and profile between the pure CS and Met-containing samples indicate altered intermolecular forces and local microenvironment. The co-existence of characteristic peaks belonging to both Met and CFZ, together with a prominent absorption band at 1555.2 cm^−1^, demonstrates successful physical incorporation of dual drugs into the CS network. As drug loading increases from C@MC_90_ to C@MC_360_, obvious shifts of peak intensity and shape are observed, which reflect network rearrangement driven by changed drug distribution and hydrogen-binding interactions rather than chemical reactions.

Since only non-covalent intermolecular interactions exist in this hydrogel system, Met and CFZ are physically entrapped within the CS network without covalent modification. This physical entrapment preserves key functional groups, namely the β-lactam ring of CFZ and guanidine moieties of Met, thus retaining their biological activity. The CS matrix offers partial shielding to stabilize the loaded drugs, and reversible hydrogen-bonding, together with electrostatic interactions, enables stimuli-responsive sustained drug release. In contrast, hypothetical covalent conjugation between drugs and CS would cause adverse risks: modification of the β-lactam ring in CFZ would eliminate its antibacterial activity, and alteration of guanidine groups of Met would impair its anti-inflammatory and antibiotic-adjuvant effects. Furthermore, covalently bound drugs would hardly diffuse out, severely compromising drug release and therapeutic efficacy. No FT-IR evidence confirms such covalent drug–CS interactions in our formulations.

As shown in Figure 1C, free CFZ exhibited a maximum absorbance at 279 nm on the ultraviolet–visible (UV-vis) spectra. After being embedded in the hydrogel and released in PBS or glucose media, the peak shifted to 313 nm (red shift), which may tentatively be attributed to molecular aggregation of CFZ molecules or intermolecular interactions between the released drug and residual CS fragments in the release medium [32]. Collectively, the FT-IR results confirm that Met and CFZ were physically incorporated into the CS matrix rather than forming new covalent chemical bonds. The peak intensity variations reflect the altered hydrogen-bonding environment inside the hydrogel network after drug loading. Such non-covalent interaction is beneficial for realizing sustained stimuli-responsive drug release without chemical modification of drug molecules, avoiding the potential chemical inactivation of β-lactam antibiotic CFZ.

### 2.3. Micromorphological Characterization via SEM

The SEM images in Figure 1D show that the CS gel possessed a smooth fracture morphology. In contrast, the Met-loaded hydrogels (C@M, Figure S1, Supplementary Materials) displayed interconnected porous structures, which favor the absorption of wound exudates and sustain a humid microenvironment at a wound site. Among these, C@M_100_ possessed the most uniform pore distribution, making it an ideal substance for dual-drug C@MC fabrication.

A series of C@MC samples (C@MC_90_, C@MC_180_ and C@MC_360_) were fabricated by introducing varied CFZ dosages into the optimized C@M_100_ substrate. Representative SEM images of C@MC hydrogels are recorded in Figure 1D, while others are shown in Figure S1. Compared with C@MC_90_ and C@MC_180_, C@MC_360_ exhibited uniform, interconnected three-dimensional pores with consistent pore size. An interconnected porous architecture is an essential prerequisite for wound healing hydrogels: open pores can absorb large amounts of wound exudate, permit gas exchange in skin tissue, and serve as a reservoir for loaded bioactive agents [19]. Well-distributed pores also facilitate diffusion of released Met and CFZ toward the wound bed, while allowing nutrient permeation for cell ingrowth. By contrast, the uneven or collapsed pore structures in C@MC_90_ and C@MC_180_ would hinder mass transportation. Therefore, C@MC_360_, with its favorable microstructure, was selected as the optimal formulation for subsequent comprehensive evaluation.

These formulation-dependent microstructural differences are consistent with previous studies on physically crosslinked CS hydrogels. Variations in the incorporated small-molecule drug amounts can modulate intermolecular supramolecular interactions and reshape the pore architecture; moderate drug addition promotes homogeneous porous networks, while insufficient or imbalanced drug loading may result in uneven or collapsed pores that hinder mass transport within hydrogel matrices [31,33].

### 2.4. Particle Size and Zeta Potential Analysis

Dynamic light-scattering (DLS)-derived hydrodynamic aggregate-size distribution serves as a critical indicator for evaluating the microstructural properties of hydrogels, which undergo obvious alterations after drug encapsulation. As depicted in Figure 1E, the pure CS sample presented with an average particle size of 5486.5 nm. Upon loading Met and CFZ, all the composite hydrogels showed different shifts in aggregate size, among which C@MC_360_ reached the maximum value of 13,774.7 nm. In addition, the drug-laden hydrogels also displayed zeta potential changes, and the zeta potential of C@MC_360_ was measured as 0.614 mV (Figure S2, Supplementary Materials). These dimensional and surface charge variations are primarily attributed to the stretching of polymer chains, the internal crosslinking density, and the introduction of new functional groups upon drug incorporation. The near-neutral zeta potential of C@MC_360_ avoids severe electrostatic repulsion or non-specific strong adsorption with negatively charged cell membranes, which is favorable for good cytocompatibility [16]. The increased hydrated particle size after dual-drug loading suggests swelling-induced polymer chain expansion, which is closely related to its drug-loading capacity and swelling behavior in wound microenvironments.

### 2.5. Rheological Behavior of Hydrogels

Conventional wound healing hydrogels generally exhibit poor mechanical properties, making them prone to destruction under external stress. Nevertheless, excessively high mechanical strength compromises hydrogel injectability. Accordingly, hydrogels suitable for wound therapy should combine mechanical strength and injectability to achieve optimal therapeutic outcomes.

The physicochemical and viscoelastic behaviors of the fabricated hydrogels could be regulated by the loading contents of Met and CFZ [31], which were evaluated through the storage modulus (G′) and loss modulus (G″). After overnight stirring, all C@MC hydrogels exhibited higher G′ values than G″ under a 1% strain and 1 Hz frequency (Figure 2A), verifying the successful formation of stable hydrogel networks. Upon gelation, the optimized C@MC_360_ exhibited an equilibrium G′ of 826.95 Pa (~0.83 kPa). This modulus continuously rose to ~14.9 kPa after 6 h of interaction (Figure 2B), as the intermolecular physical bonding continuously promoted the progressive construction of hydrogel networks. The shear viscosity depended positively on the CFZ dosage. Notably, C@MC_360_ displayed prominent shear-thinning properties under shear rates ranging from 0.1 to 100 s^−1^ (Figure 2C), serving as an essential feature for injectable hydrogel applications.

Alternate strain cycles (1–100%) were performed to assess the self-healing capability (Figure 2D). At 100% strain, the hydrogel network broke down and G′ < G″. Once the strain returned to 1%, the network rapidly recovered, with G′ > G″. Such reversible structural transitions endow C@MC with injectability and structural stability for wound treatment. Furthermore, three loading–unloading cycles at 70% strain further manifested the favorable resilience and self-healing capacity of C@MC_360_ (Figure 2E).

Overall, the advantages of enhanced mechanical properties, compressional resilience, injectability and self-healing render C@MC a promising candidate biomaterial for wound dressings. The equilibrium storage modulus (~0.83 kPa) of C@MC_360_ falls within the mechanical modulus window reported for skin-contact injectable CS hydrogel dressings [16]. An overly high modulus leads to poor compliance against deformed/moving skin, whereas insufficient mechanical strength causes rapid hydrogel disintegration upon exudate immersion. The observed shear-thinning property enables syringe extrusion, and the strain-reversible self-healing behavior allows the hydrogel to reconstruct the network after mechanical destruction at a wound site. These rheological features collectively guarantee the conformal adaptation of the hydrogel to dynamically changing wound surfaces, such as joint skin.

### 2.6. Drug Release Assay

#### 2.6.1. Standard Curve

The UV–vis absorbances of serially diluted Met solutions were measured at 232 nm. The standard curve of Met is shown in Figure S3A (Supplementary Materials). The linear fitting equation is A = 0.08635C + 0.30172, accompanied by R^2^ of 0.99975. The absorbance values of different prepared CFZ solutions were determined at an absorption wavelength of 279 nm. The CFZ standard curve is shown in Figure S3B (Supplementary Materials). The linear fitting equation is A = 0.01662C + 0.0501, with R^2^ calculated to be 0.99639. A is the absorbance of the measured solution, and C stands for the solution concentration (μg/mL).

#### 2.6.2. CFZ Release

Figure 2F illustrates the release profiles of C@MC_360_ in PBS at different pH conditions, while the release behaviors of C@MC_90_ and C@MC_180_ are displayed in Figure S3C,E. Furthermore, the drug release behavior of C@MC_360_ in glucose solutions with varying concentrations is shown in Figure 2G. The corresponding release data of C@MC_90_ and C@MC_180_ groups are provided in Figure S3D,F.

The cumulative CFZ release increased progressively over time regardless of drug loading or release media, and the release rate was significantly influenced by the pH and glucose content. Faster drug release was observed under mildly acidic and high-glucose conditions. Under acidic surroundings, protonation of CS amino groups weakens intramolecular hydrogen bond crosslinking and loosens the hydrogel network. Excess glucose competes for hydrogen-bonding sites between CS and the encapsulated drugs, further facilitating drug diffusion from the matrix. C@MC_360_ achieved sustained release of both CFZ and Met, with >85% of the total drug released by day 9, followed by sustained slow release up to day 15. This time-dependent, environment-responsive release enables long-term controllable drug output under wound-relevant conditions. Notably, although wound beds generally exhibit a neutral-to-alkaline bulk pH, localized acidic micro-domains arise at foci of active bacterial infection. The accelerated release under acidic conditions therefore represents responsiveness to these infection-associated micro-niches, while moderate sustained drug release is still preserved at neutral physiological pH. Such stimulus-triggered drug liberation is advantageous over non-responsive hydrogels with unregulated constant drug leakage.

#### 2.6.3. Met Release

Figure S3G,H show the cumulative Met release profiles of the C@MC_360_ in PBS with various pH values and glucose solutions of different concentrations. Met exhibited continuous cumulative release alongside the incubation time in PBS at pH 4.4, 5.4, 6.4 and 7.4. Samples immersed in lower pH conditions of 4.4 displayed accelerated drug release, reaching a total release of 75.89%. The release rate decreased successively as the pH rose to neutral 7.4.

Met also demonstrated continuous release in glucose solutions of 5, 10 and 50 mM. Overall, a higher glucose content generally promoted quicker drug release. This tendency (faster release under mildly acidic conditions and slower release at near-neutral pH) indicates that the hydrogel enables sustained and controlled drug release at a wound site.

### 2.7. Adhesion and Injectability

An ideal hydrogel dressing should exhibit good adhesiveness to ensure full contact with the wound surface to prevent infection. Figure 2I verifies that the C@MC adhered stably to skin and glass surfaces. No detachment was observed when C@MC was applied on finger joints and flexed pig skin. The hydrogel retained its complete structure and closely covered the skin tissue. Unlike traditional hydrogels, injectable self-healing hydrogels are well suited for covering irregular wound sites and resisting infection.

C@MC_360_ was selected as the representative sample for injectability verification. As direct experimental proof of injectability, C@MC_360_ could be smoothly and continuously extruded from a syringe to form the word “gel”. After the shear stress was removed, the extruded material retained a stable gel state at room temperature for an extended period (Figure 2H). This macroscopic observation is consistent with the shear-thinning and rapid self-healing rheological characteristics described in Section 2.5, where the reversible supramolecular interactions were shown to endow the hydrogel with injection capability.

These results confirm that the C@MC hydrogel combines favorable injectability and tissue adhesion. The hydrogel fits wounds with diverse shapes and reveals promising prospects for advanced wound dressing applications. Good tissue adhesion prevents premature dressing delamination from irregular wound surfaces without extra adhesive tape fixation. Combined with injectability, C@MC hydrogel can fill deep, irregular infected wound defects, a prominent advantage over traditional pre-formed hydrogel sheets.

A moderately moist microenvironment supports fibroblast function and wound regeneration, yet excess moisture can facilitate bacterial colonization [14,34]. Thanks to its interconnected porous architecture, the C@MC hydrogel absorbs excess wound exudates and prevents wound over-hydration. Meanwhile, its local bactericidal activity offsets the risk of bacterial proliferation associated with moist wound -healing conditions.

### 2.8. Antibacterial Properties

Effective antibacterial activity is essential for wound dressings applied to infected lesions. Systematic antibacterial assessments were performed to characterize the inhibitory capacity of C@MC hydrogel against *Staphylococcus aureus* (*S. aureus*), *Escherichia coli* (*E. coli*) and NDM-1 *E. coli*. Figure 3A–C show that the antibacterial activity of C@MC changed with the hydrogel concentration, and all C@MC groups generated remarkable bacterial suppression compared to the blank controls.

C@MC_180_ achieved antibacterial inhibition rates of 95.3%, 90.8% and 95.3% against the three bacterial strains, while the corresponding values of C@MC_360_ were 95.1%, 91.1% and 94.1%. The untreated control group yielded abundant bacterial colonies. However, no colonies survived after C@MC_360_ treatment, confirming its complete bactericidal activity. In contrast, CFZ alone and single-Met C@M_100_ showed limited antibacterial outcomes, with inhibition rates correspondingly ranging from 24.2 to 39.5% and 37.6 to 90%. These findings demonstrate that combined delivery of CFZ and Met within the C@MC hydrogel produced markedly enhanced bactericidal activity against the test bacteria, including NDM-1 *E. coli*. Met functions as an antibiotic adjuvant to improve the antibacterial performance of CFZ.

A live/dead bacterial staining analysis (Figure 3E) further characterized the effects of different treatments on bacterial viability. The bacteria in the control and C@gel groups emitted strong green fluorescence, reflecting high survival rates. Notably, this potent antibacterial action was equally pronounced against drug-resistant NDM-1 *E. coli*, with a killing rate higher than 90%. The SEM images in Figure 3G offer direct morphological evidence of bacterial structural damage. The bacteria in the C@MC_180_ and C@MC_360_ groups show a sharp reduction in quantity, along with obvious cell deformation, rupture and shrinkage. Plate colony counting and live/dead staining validate the complete bacterial elimination by C@MC_360_, benefiting from its sustained long-term drug release rather than transient short-term inhibition.

As shown by the antibacterial results, CFZ alone displayed a limited killing effect toward NDM-1 *E. coli*. This can be explained by the NDM-1 metalloenzyme that hydrolyzes the β-lactam ring and deactivates CFZ. The single Met group also could not realize complete bacterial elimination. Only co-delivery of Met and CFZ achieved prominent synergistic bactericidal performance. Consistent with previous drug-repurposing research [24,29], Met acts as an antibiotic adjuvant to reverse β-lactam resistance. It may disturb bacterial membrane homeostasis or interfere with the NDM-1-related resistance pathway, restoring susceptibility of resistant bacteria to CFZ. The bacterial membrane rupture and shrinkage observed in the SEM further confirms the damage to bacterial integrity induced by this synergistic effect. The sustained drug release from the porous hydrogel matrix contributed to a long-lasting bactericidal effect rather than transient inhibition.

To rule out interference from the hydrogel acidity, all in vitro antibacterial tests were carried out within buffered LB medium, which offset the initial acidic pH of freshly prepared hydrogels. Therefore, the observed bactericidal activity derived from the synergistic effect of metformin and cefazolin, instead of acid-mediated bacterial inhibition.

### 2.9. In Vitro Compatibility and Anti-Inflammatory Effect of C@MC

Biocompatibility serves as a key requirement for bioactive hydrogels designed to be used in clinical wound dressing. An MTT assay combined with live/dead staining was adopted to evaluate compatibility of these hydrogels. According to Figure 4A–E, C_2_C_12_ cells incubated with C@MC hydrogel extracts for 24 h showed no obvious changes in cell viability, which remained above 90% in all treatment groups. The Live/dead staining assays (Figure 4G) further verified the aforementioned results. Most C_2_C_12_ cells treated with the C@MC hydrogel maintained a typical spindle-shaped adherent morphology, with over 85% of cells staying intact. Green fluorescence from the viable cells prevailed, and the dead cells emitting red fluorescence only accounted for less than 15%. It can be concluded that the C@MC hydrogel possesses negligible toxicity and satisfactory biocompatibility.

Malondialdehyde (MDA) measurements (Figure 4F) were carried out to quantify the cellular lipid peroxidation damage and indirectly evaluate the cellular oxidative stress. Quantitative MDA detection revealed levels of C@MC_90_ and C@MC_180_ groups were 0.0387 and 0.0375 μM, with reduction rates of 16.0% and 18.7%. Both values were lower than the 0.0461 μM measured in the H_2_O_2_-treated control group. These results indicate that the drug-loaded hydrogel can scavenge intracellular ROS and mitigate oxidative cellular injury.

Excessive ROS accumulation in an infected wound drives lipid peroxidation and generates mass amounts of MDA. This process further activates the NF-κB signaling pathway in macrophages, thereby stimulating the secretion of pro-inflammatory TNF-α and IL-6. This pathological interplay forms a self-sustaining vicious cycle between oxidative stress and persistent inflammation. By continuously eliminating intracellular ROS and limiting MDA accumulation, the dual-drug C@MC hydrogel may suppress upstream NF-κB pathway activation and consequently reduce pro-inflammatory mediator release from macrophages. The antioxidant and anti-inflammatory functions of C@MC are not independent effects, and they operate to remodel the oxidative–inflammatory microenvironment of damaged skin.

An effective anti-inflammatory capability is vital for hydrogels to avoid abnormal inflammation during wound healing. To assess the anti-inflammatory effect of C@MC, an inflammatory cell model was built using RAW 264.7 macrophages induced by LPS. Two typical pro-inflammatory cytokines, TNF-α and IL-6, were detected to comprehensively analyze the anti-inflammatory capacity of the C@MC hydrogel. Relative to the LPS control group, the C@MC_360_ groups obviously suppressed the expression of the two cytokines (Figure 4H,I). The concentration of TNF-α fell by 57.41 pg·mL^−1^, and IL-6 decreased by 15.63 pg·mL^−1^. On this basis, the inhibition efficiencies of TNF-α and IL-6 reached 30.9% and 33.8%. Furthermore, even compared with the CFZ group, C@MC_360_ still exhibited approximately 10.1% and 3.7% higher anti-inflammatory activity toward TNF-α and IL-6, respectively. In conclusion, the C@MC composite hydrogel integrates potent antibacterial activity, excellent biocompatibility and antioxidant capacity, and remarkable anti-inflammatory activity, rendering it an ideal multifunctional candidate for advanced wound dressing development.

In infected wound sites, bacterial invasion induces excessive ROS production. It should be noted that physiological ROS mediates host antimicrobial defense. We envision that the hydrogel preferentially scavenges pathological excess ROS to mitigate oxidative injury, without abolishing the physiological ROS required for host defense [35]. Uncontrolled ROS buildup triggers lipid peroxidation and MDA generation, which further activates the NF-κB signaling pathway in macrophages and promotes secretion of pro-inflammatory TNF-α and IL-6, forming a vicious oxidative–inflammatory feedback loop [26]. Our in vitro data demonstrate that the C@MC hydrogel disrupts this pathological cascade: the Met-containing hydrogel scavenges ROS, decreases MDA levels and suppresses pro-inflammatory mediator release. Notably, this anti-inflammatory activity is not attributable merely to CFZ-mediated bacterial killing; comparisons against the CFZ-only group reveal that Met confers additional immunomodulatory effects. Favorable cytocompatibility guarantees that the hydrogel does not introduce extra cytotoxicity to endogenous skin wound cells. It is worth noting that TNF-α and IL-6 are representative pro-inflammatory markers. Wound inflammation is governed by a complex cytokine network and cannot be comprehensively assessed using only these two readouts.

### 2.10. Full-Thickness Wound Healing Ability of C@MC

We first constructed an *E. coli*-infected full-thickness wound mouse model as a reference to contrast the therapeutic performance of hydrogel against clinically threatening NDM-1 *E. coli.* Five hydrogel formulations, including C@gel, C@M_100_, C@MC_90_, C@MC_180_ and C@MC_360_, were included in the comparative experiments. For administration, 0.2 g of each hydrogel sample was directly injected to cover the wound surface, then secured with sterile gauze. The wound healing status was monitored at different time intervals according to the experimental model. Comprehensive evaluations were performed on day 14 via gross visual observation and histopathological analysis (Figure 5D,E).

The macroscopic images confirmed faster wound closure in the hydrogel-treated groups versus blank controls (Figure 5A). The C@MC_360_ achieved the highest healing efficiency throughout the experiment. By day 14, the blank group still retained a residual wound area of 20.68% (Figure 5B). All C@MC treatment groups attained nearly full wound closure, with residual areas ranging from 5.19% to 10.64%. Notably, C@MC_360_ yielded the lowest residual area of merely 5.19%. The data proves the composite hydrogel can greatly speed up wound regeneration.

The quantitative immunohistochemical data (Figure 5C,E) showed TNF-α and IL-6 positive expression above 20% in the control and C@gel wound tissue, proving these treatments poorly optimized the wound inflammatory microenvironment. On the contrary, the positive expression rates of the two inflammatory cytokines in the C@MC_90_, C@MC_180_ and C@MC_360_ groups were significantly downregulated. Among them, the C@MC_360_ group achieved the greatest inhibitory effect, with the positive expression rates of IL-6 and TNF-α reduced to 5.57% and 7.21%, respectively.

In conclusion, the C@MC composite hydrogel can optimize the wound healing microenvironment and then accelerate the process of tissue regeneration by efficiently inhibiting the local inflammatory response of a wound. Moreover, this effect presents a certain dose-dependent pattern, with the C@MC_360_ dose being the optimal one for promoting wound healing among this series of hydrogels.

The hematoxylin–eosin (H&E) staining results further confirmed the previous macroscopic results (Figure 5D). The histology revealed incomplete re-epithelialization in the control wounds, alongside diffuse dermal infiltration of lymphocytes and neutrophils, plus irregular, loose granulation tissue. However, all hydrogel-treated groups showed alleviated inflammatory infiltration, tightly arranged granulation tissue, complete epithelial regeneration and a well-organized tissue structure on day 14. The HE-stained sections demonstrated remarkable decreases in neutrophils (yellow arrows), lymphocytes (green arrows), plasma cells (blue arrows), and macrophages (red arrows) within the hydrogel-treated wounds (Figure 5D). Among these groups, the C@MC_360_ hydrogel achieved the optimal tissue repair effect, verifying its ability to modulate inflammation and promote tissue regeneration and remodeling. The in vivo healing tendency was highly consistent with our in vitro findings. Compared with the blank CS gel without drugs, the C@MC series relieved excessive inflammatory infiltration, which may be attributed to the combined synergistic antibacterial, antioxidant and anti-inflammatory functions. Effective elimination of invading bacteria reduces the initial infection stimulus, while local ROS clearance and pro-inflammatory factor inhibition create a pro-regenerative microenvironment, promoting granulation tissue maturation and re-epithelialization. Among all formulations, C@MC_360_ exhibited the best therapeutic outcome, which agreed with its optimal physicochemical properties and in vitro bioactivity.

### 2.11. Full-Thickness Wound Healing Ability of C@MC Against NDM-1 E. coli Infection

Based on the promising wound-repair outcomes obtained from the above-mentioned *E. coli*-infected model, we further built a separate NDM-1 *E. coli*-infected full-thickness wound model using another independent cohort of KM mice. This model served to assess the therapeutic effects against clinical drug-resistant bacterial wound infections.

The quantitative wound area statistics (Figure 6A,B) revealed an only 4.2% residual wound area in the C@MC_360_ group at day 14, far lower than the 18.7% residual area of blank control. To further clarify the regulatory effects of the C@MC hydrogel on tissue regeneration in bacterially infected wounds, systematic histological analyses were performed on day 14, including H&E staining (Figure 6D) and immunohistochemical staining (Figure 6E). The H&E histological observations revealed massive infiltration of inflammatory cells, disordered tissue structure and loose granulation tissue in the control wound beds. In the H&E-stained sections (Figure 6D), neutrophils and lymphocytes are indicated by yellow and green arrows, respectively. In comparison, all C@MC hydrogel-treated groups showed intact epidermal integrity, well-organized dermal tissue architecture, and significantly improved maturity of newly regenerated tissues. Among these groups, the C@MC_360_ group exhibited the most-ordered tissue regeneration, indicating that the composite hydrogel can effectively optimize the microenvironment of infected wounds and facilitate their orderly regeneration.

The immunohistochemical quantification of IL-6 and TNF-α (Figure 6C) revealed positive rates of 21.74% and 23.68% in the control group, which illustrate that NDM-1 *E. coli* infection provoked severe inflammatory reactions at the wound sites and greatly slowed wound healing progress. The C@MC_90_, C@MC_180_ and C@MC_360_ groups all obviously inhibited the expression of the above inflammatory factors. Meanwhile, the C@MC_360_ displayed superior inhibitory capacity, with the positive expression rates of IL-6 and TNF-α reduced to 5.63% and 7.91%, respectively.

In particular, the C@gel group also exhibited evident wound healing capacity, consistent with the well-documented intrinsic bioactivity of CS, which promotes wound repair by maintaining a moist microenvironment, modulating inflammation and facilitating cell migration [36]. Although the macroscopic wound closure rates showed limited statistical differences across hydrogel groups, C@MC_360_ achieved the best overall performance. It displayed stronger antibacterial activity against NDM-1 *E. coli*, relieved oxidative inflammation and improved granulation tissue formation and collagen deposition. These improvements represent enhanced wound quality and infection control, rather than accelerated wound closure.

Clinically, prolonged topical antibiotic administration is generally avoided for chronic wounds because it may promote resistance among wound microbes, including unculturable strains; antiseptics are typically preferred for routine topical wound disinfection [37,38]. Distinct from conventional long-term topical antibiotic therapy, our dual-drug hydrogel uses Met as an antibiotic adjuvant to restore β-lactam susceptibility in resistant bacteria, enabling efficient bacterial clearance within a relatively short treatment window and shortening the antibiotic exposure time. Still, the risk of resistance selection by locally delivered CFZ cannot be completely ruled out. Further preclinical studies, including polymicrobial infection models, are needed to evaluate resistance evolution and better recapitulate native wound microbiomes prior to clinical translation.

## 3. Conclusions

In summary, this work reports the fabrication of an injectable multifunctional CS-based dual-drug hydrogel, C@MC, co-loaded with Met and CFZ for combating drug-resistant bacteria wound infections. The optimized C@MC_360_ formulation forms interconnected three-dimensional porous networks with a G′ of approximately 0.83 kPa, affording suitable mechanical properties, injectability, and tissue adhesion for treating irregular wound defects. Owing to the adjuvant-assisted combined antibacterial effect from Met and CFZ, the hydrogel achieves potent concentration-dependent bactericidal effects against *S. aureus*, *E. coli* and NDM-1 *E. coli*. In addition to its antibacterial performance, C@MC effectively scavenges ROS, lowers cellular MDA levels, and suppresses pro-inflammatory TNF-α and IL-6 release from LPS-activated macrophages. In the in vivo full-thickness wound model challenged with NDM-1 *E. coli*, C@MC_360_ markedly mitigated inflammatory infiltration, promoted re-epithelialization and ordered tissue remodeling, and yielded a mere 4.2% residual wound area on day 14.

This work repurposes the clinically used oral hypoglycemic agent Met as a bioactive component in wound healing biomaterials, providing a feasible multifunctional strategy for skin wounds infected with drug-resistant bacteria. Of note, our study relies on a single-species infected wound mouse model, which fails to recapitulate hallmarks of human chronic wounds, such as polymicrobial flora, hypoxia and host metabolic disorders. Moreover, immortalized mouse cell lines instead of primary or human-derived skin cells were used for the in vitro assays. Further validation using polymicrobial infection models, diabetic wound models and human-relevant primary skin cells is therefore required for clinical translation. In addition, the precise molecular interactions between Met and NDM-1 metalloenzyme remain incompletely elucidated. Future mechanistic work will decipher these molecular events and quantitative drug–drug interactions to guide the rational design of next-generation anti-resistance wound dressings.

## 4. Materials and Methods

### 4.1. Materials

All the reagents utilized in the experiments were acquired from Shanghai Aladdin Biochemical Technology Co., Ltd. (Shanghai, China). The commercial assay kits used in this work were purchased from Thermo Fisher Scientific Inc. (Waltham, MA, USA). Standard strains, including Gram-negative bacterium *E. coli* (ATCC 25922) and Gram-positive bacterium *S. aureus* (ATCC 6538), were supplied by the China Center of Industrial Culture Collection (Beijing, China). The NDM-1 *E. coli* was kindly provided by Professor Kewu Yang from Northwest University. The mouse C_2_C_12_ cells, RAW 264.7 macrophages and all antibodies were obtained from Wuhan Servicebio Technology Co., Ltd. (Wuhan, China).

### 4.2. Preparation of C@M and C@MC

Met hydrogels (C@M) were synthesized via one-pot approach. CS (100 mg, deacetylation degree ≥ 90%) was dispersed in acetic acid solution (0.1 M, 4 mL) and stirred for 2 h at room temperature to full dissolution. Met (10, 50, 100 mg) was supplemented and stirred for additional 12 h. Gelation was confirmed by rheological crossover point (G′ > G″). Monometformin hydrogels were denoted as C@M_10_, C@M_50_, C@M_100_. Based on pre-screening of gelling behavior, microstructure, antioxidant and anti-inflammatory performance, 100 mg Met (C@M_100_) was selected for fabricating dual-drug C@MC hydrogels.

Appropriate CFZ was mixed into pre-formed C@M_100_ sol and magnetically stirred for 12 h at room temperature to achieve homogeneity. Sol was transferred to glass vials and statically incubated for 6 h at room temperature without extra crosslinkers or post-treatment to form bulk hydrogel (gelation process shown in Figure 1A). Gelation time was recorded (*n* = 3). System pH was maintained at 4.2–4.6 by 0.1 M acetic acid; Met and CFZ induced negligible pH shifts. Hydrogel equilibrated to physiological wound pH upon exposure to wound exudates.

C@MC_90_, C@MC_180_ and C@MC_360_ correspond to 90, 180, and 360 μg CFZ per 100 mg CS matrix, with fixed Met dosage (100 mg) and Met/CFZ molar ratios of 16:1, 8:1 and 4:1. Gelation originates from supramolecular physical crosslinking (hydrogen bonding and electrostatic interactions) between CS and drug molecules. This physical encapsulation preserves β-lactam ring of CFZ without covalent chemical modification.

### 4.3. Electron Microscopic Characterization of C@MC

Hydrogel samples were frozen in liquid nitrogen and fractured for cross-section observation, followed by pre-freezing (−80 °C, 4 h) and vacuum freeze-drying (−55 °C, 12 h) as previously described [31]. Dried samples were sputter-coated with 10 nm platinum and imaged by field-emission scanning electron microscopy (FESEM, MCLARA, TESCAN, Brno, Czech Republic) at accelerating voltage of 5 kV.

### 4.4. Infrared Spectrum of Hydrogel

In terms of hydrogel samples, after being frozen in liquid nitrogen, the freshly prepared specimens were kept at −80 °C for 4 h and further lyophilized for 12 h to obtain the dry samples for FT-IR testing [39]. The spectra were scanned over a wavenumber range of 4000–400 cm^−1^ using a Nicolet iS50 spectrometer (Thermo Fisher, Waltham, MA, USA).

### 4.5. Zeta Potential and Particle Size of C@MC Hydrogel

The dried gel sample (50 mg) was immersed in 10 mL PBS and soaked until a swelling equilibrium was reached. Aliquots of 1 mL were taken at each time, and the zeta potential and particle size were measured at room temperature by a zeta potentiometer (Nano-25, Malvern Panalytical, Malvern, UK). All measurements were performed in triplicate.

### 4.6. Rheological Properties of C@MC

Circular splines with 25 mm diameters were trimmed from the bulk hydrogel samples, and rheological characterization was carried out at 25 °C via a rheometer (MCR 92, Anton Paar, Graz, Austria). Dynamic strain sweep assays were adopted to identify the linear viscoelastic region of all gel samples. During the strain sweep tests, the angular frequency was kept constant at 10 rad/s, and the shear strain was gradually varied from 0.1% to 100%. Under the constant shear strain condition at γ = 0.5%, the rheological tests were conducted within an angular frequency interval range of 0.01 to 100 rad/s.

### 4.7. Study on Drug Release of C@MC

PBS buffers with pH gradients of 7.4, 6.4, 5.4 and 4.4, alongside glucose solutions at 5, 10 and 50 mM, were prepared at room temperature to serve as the release media for the drug delivery tests. Briefly, weighted hydrogel samples were sealed inside dialysis bags (molecular weight cutoff = 8–14 kDa; Viskase, Lombard, IL, USA) [40]. Each dialysis bag was immersed in 50 mL of corresponding release medium inside sealed Erlenmeyer flasks placed in a constant-temperature shaking incubator (THZ-82A, Jintan Instrument, Jintan, China) maintained at 37 °C with continuous oscillation at 120 rpm. C@M_100_ (100 mg) was loaded into dialysis bags to monitor the release of Met. Separately, 550 mg each of C@MC_90_, C@MC_180_ and C@MC_360_ was weighed and sealed in dialysis bags, and then immersed in 10 mL of PBS buffer solution and glucose solution to monitor the release of CFZ.

Sink conditions were maintained during the whole experiment. CFZ has high aqueous solubility (>100 mg/mL). Even for the highest-loading C@MC_360_ group, the theoretical maximum CFZ concentration after complete drug release into 10 mL of medium was only 36 μg/mL, well below its saturation level. At predetermined time points (day 0, 1, 3, 5, 7, 9, 12 and 15), 2 mL of supernatant was collected, followed by immediate replenishment with an equal volume of pre-equilibrated fresh medium [34]. Periodic medium replacement kept the drug concentrations below 20% of saturation. A 50 mL medium volume was adopted for Met release to ensure sink conditions. Drug concentrations were measured via a UV-vis spectrophotometer (Cary 3500, Agilent Technologies, Lexington, MA, USA) [41], with detection wavelengths set at 232 nm for Met and 279 nm for CFZ.

The cumulative amount of released drug at each time point (*M_t_*) was calculated via the following formula:

$$M_{t}=C_{n}V+\sum_{i=1}^{n-1}C_{i}V_{s}$$

where *C_n_* represents the drug concentration of the nth sample, *V* is the total volume of the release medium, *V_s_* is the sampling volume at each time point, and *C_i_* represents the drug concentration of the *i*th sample. The cumulative drug release percentage was then calculated using the following formula:

$$\mathrm{Cumulative}\;\mathrm{Release}\;(\%)=\frac{M_{t}}{M_{0}}\times 100\%$$

### 4.8. Adhesion and Injectability of C@MC

The adhesion performance was evaluated by spreading the C@MC hydrogel onto ex vivo porcine skin, articular tissue and glass substrates, followed by static incubation to observe spontaneous detachment. For injectability testing, the hydrogel was loaded into 10 mL syringes and extruded onto black cardboard to examine the homogeneous outflow.

### 4.9. In Vitro Antibacterial Activity Test of C@MC

All test strains (*S. aureus*, *E. coli* and NDM-1 *E. coli*) were cultivated in LB medium at 37 °C for 12 h, then diluted with sterile PBS to 1 × 10^8^ CFU·mL^−1^ for subsequent antibacterial assays [42]. All in vitro antibacterial assays were performed with *n* = 3 independent biological replicates. Quantitative data were expressed as mean ± SD.

#### 4.9.1. Bacterial Growth Viability Monitoring and Bacterial Plate Counting

Standardized bacterial suspension was diluted 100-fold, and 5 mL bacterial diluent was incubated with PBS or 0.2 g hydrogel in 12-well plates. Bacterial growth was monitored by measuring OD_600_ at predetermined time points (0, 2, 4, 6, 8, 12, and 24 h) using microplate reader (M2, Meggu Molecular Instruments Co., Ltd., Los Angeles, CA, USA). For colony counting, 10 μL bacterial suspension was incubated with hydrogels (37 °C, 2 h). Viable bacteria were resuspended with sterile PBS, diluted 100-fold, and 100 μL of suspension was spread onto agar plates. After incubation (37 °C, 14 h), colony numbers were counted. PBS-treated bacterial suspension served as control and underwent identical processing.

#### 4.9.2. Bacterial Live/Dead Staining

The effect of each gel on the bacterial survival rate was evaluated using a bacterial live/dead staining kit. The bacterial suspension was inoculated into sterile petri dishes containing C@gel, C@M_100_, C@MC or CFZ. The plates were incubated at 37 °C for 24 h. Subsequently, a 3,6-diamidino-2-phenylindole/propidium iodide (DAPI/PI) staining solution prepared according to the kit instructions was added, followed by incubation in a dark box for 15 min. Finally, observation and photography were performed using an inverted fluorescence microscope (A1R HD25, Nikon Corporation, Tokyo, Japan) to record the results.

#### 4.9.3. Scanning Electron Microscopy of Bacteria

Each hydrogel sample (5 g) was coincubated with 50 mL of corresponding bacterial suspension overnight at 4 °C. Residual gel debris was removed by low-speed centrifugation (1500 rpm, 3 min). Bacterial pellets were harvested (5000 rpm, 8 min; Avanti J-15/15R centrifuge, Beckman Coulter, Inc., Indianapolis, IN, USA), washed three times with sterile PBS (5 mL), fixed overnight in 2% glutaraldehyde (4 °C), and subjected to graded ethanol dehydration (30%, 50%, 70%, 90%, 100%, *v*/*v*) with 10 min for each step. Samples were treated with isoamyl acetate (4 °C, 30 min), critical-point dried and sputter-coated prior to SEM imaging.

### 4.10. Cytocompatibility of C@MC

C_2_C_12_ mouse myoblasts were used to assess cytocompatibility via MTT assay and live/dead cell fluorescence staining. Hydrogels were immersed in 5 mL of serum-free DMEM (solid–liquid ratio, 100 mg/mL) and shaken at 37 °C for 24 h to prepare hydrogel extracts.

For MTT assay, C_2_C_12_ cells (5 × 10^4^ cells/well) were seeded in 96-well plates and cultured (37 °C, 5% CO_2_) until attachment. Cells were incubated with serially diluted hydrogel extracts for 24 h. MTT reagent (0.5 mg/mL) was added and incubated for 4 h. After removing medium, 150 μL DMSO was added to dissolve formazan crystals (10 min gentle shaking). Absorbance at 490 nm was measured using microplate reader (TC20, Bio-Rad, Hercules, CA, USA). Serum-free medium served as control; cell-free medium was set as blank. Cell viability was calculated as the following formula:

$$\mathrm{Cell}\;\mathrm{viability}\;\%=\frac{A_{s}-A_{b}}{A_{c}-A_{b}}\times 100\%$$

In formula, A_s_, A_b_ and A_c_ denote absorbance values of sample, blank group, and control group, respectively.

For live/dead staining, C_2_C_12_ cells (5 × 10^4^ cells/well in 96-well plates) were treated with 5 mg/mL hydrogel extract for 24 h. Fluorescence images were acquired under inverted fluorescence microscope.

### 4.11. Antioxidation Assays for C@MC

#### 4.11.1. Screening of H_2_O_2_ Concentration

C_2_C_12_ cells (5 × 10^4^ cells/well) were seeded in 96-well plates and cultured (37 °C, 5% CO_2_, 24 h) for cell attachment. Serially diluted H_2_O_2_ (0–1000 μM in serum-free medium) was added and incubated for 24 h to induce oxidative damage. After PBS washing, MTT reagent (10 μL, final concentration 0.5 mg·mL^−1^) was added for 4 h incubation. Medium was removed, 150 μL DMSO was added, and plates were shaken gently for 10 min to dissolve formazan crystals. Absorbance at 490 nm was measured by microplate reader. H_2_O_2_ concentration producing approximately 50% cell viability was chosen for subsequent oxidative injury experiments.

#### 4.11.2. Evaluation of Antioxidant Capacity

C_2_C_12_ cells (5 × 10^5^ cells/well) were seeded in 6-well plates and cultured for 24 h to achieve full adherence. Cell monolayers were treated with H_2_O_2_ working medium (2 mL, 24 h) to establish oxidative-stress injury. After H_2_O_2_-containing medium was removed, cells were rinsed once with PBS and incubated with 2 mL of respective hydrogel leachate for another 24 h. Cells were then collected and lysed to collect soluble supernatants. Intracellular MDA levels were quantified using MDA assay kit. Obtained data were statistically analyzed to compare antioxidant protection among hydrogel groups.

### 4.12. Anti-Inflammatory Experiment on C@MC

The anti-inflammatory ability of prepared hydrogels in vitro was evaluated using a lipopolysaccharide (LPS) inflammatory cell model based on RAW 264.7 macrophage. After seeding at 5 × 10^4^ cells/well in 96-well plates, the cells were treated with 100 μL of 1 μg·mL^−1^ LPS to trigger inflammatory activation. The LPS-induced inflammatory model was validated by measuring the elevated NO concentration in cell supernatant via a NO detection kit. After 24 h of cell adhesion, 100 μL hydrogel extract was added for another 24 h incubation. The cell culture supernatants were collected to detect the levels of pro-inflammatory factors, including TNF-α and IL-6, via commercial enzyme-linked immunosorbent assay (ELISA) kits.

### 4.13. Full-Thickness Wound Healing Experiment

Male KM mice (8–12 weeks old, 35–40 g) were used to establish two independent sets of full-thickness infected wound models: one infected with standard *E. coli* and the other infected with NDM-1 *E. coli*. All experimental animals received a one-week acclimatization period under standardized breeding conditions (12 h light/dark cycle, stable temperature and free access to regular diet and drinking water) before modeling, and individuals with physical injuries or illness were eliminated in advance. For the surgical operation, the mice received an intraperitoneal injection of tribromoethanol (250 mg/kg) to achieve adequate anesthesia. The dorsal hair of each mouse was shaved with a razor, and a circular full-thickness skin defect with a diameter of 8 mm was generated on the dorsal midline. For bacterial infection modeling, 40 μL bacterial suspension (1 × 10^8^ CFU·mL^−1^) was uniformly applied to the fresh wound surface. The wounds were then covered with sterile gauze for 24 h to facilitate stable bacterial colonization.

The experimental animals were randomly assigned to seven groups with *n* = 8 biological replicates for each group, consisting of blank control, C@gel, pure CFZ, C@M_100_, C@MC_90_, C@MC_180_, and C@MC_360_. The mice in the CFZ group were treated with 100 μL CFZ solution (500 μg·mL^−1^), while each hydrogel group was directly placed to fully cover the wound with 200 mg sterilized composite hydrogel. All dressings were fixed with medical gauze, and the mice were individually caged to prevent mutual scratching and wound damage. The hydrogel and gauze dressings were replaced daily. Before each dressing change, 100 μL PBS was applied to reduce adhesion and prevent additional wound trauma. The mice were euthanized on day 14 post-hydrogel administration. The wounds were photographed, and their areas were measured via Image J to calculate the wound closure ratios. The repaired skin tissues were excised for histological analysis [33].

### 4.14. Histological Analysis

Day 14 wound tissues were subjected to H&E staining and immunohistochemistry for evaluation of wound regeneration and detection of IL-6 and TNF-α expression, following previously described protocols [32]. Tissue sections were incubated overnight at 4 °C with primary antibodies targeting TNF-α (Cat. No. GB115702, Servicebio Technology, 1:1000 dilution) and IL-6 (Cat. No. GB11117, Servicebio Technology, 1:300 dilution). Sections were then incubated with HRP-conjugated goat anti-rabbit secondary antibody (Cat. No. G1302, Servicebio Technology) for 50 min at room temperature. 3,3′-diaminobenzidine (DAB) chromogenic reaction was performed for 5 min, followed by hematoxylin counterstaining for 3 min. After staining, all tissue sections were dehydrated, cleared and mounted. Microscopic imaging was conducted, and immunohistochemical positive signals were quantified using Image J software.

### 4.15. Statistical Analysis

All quantitative data were expressed as mean ± standard deviation (mean ± SD). Biological replicate number n is stated in corresponding figure captions. One-way analysis of variance (ANOVA) followed by Tukey’s multiple comparisons post hoc test was performed for multi-group comparisons. Student’s *t*-test was applied for two-group comparisons. All statistical analyses were performed using GraphPad Prism 9 software.

## Figures and Tables

**Figure 1:**
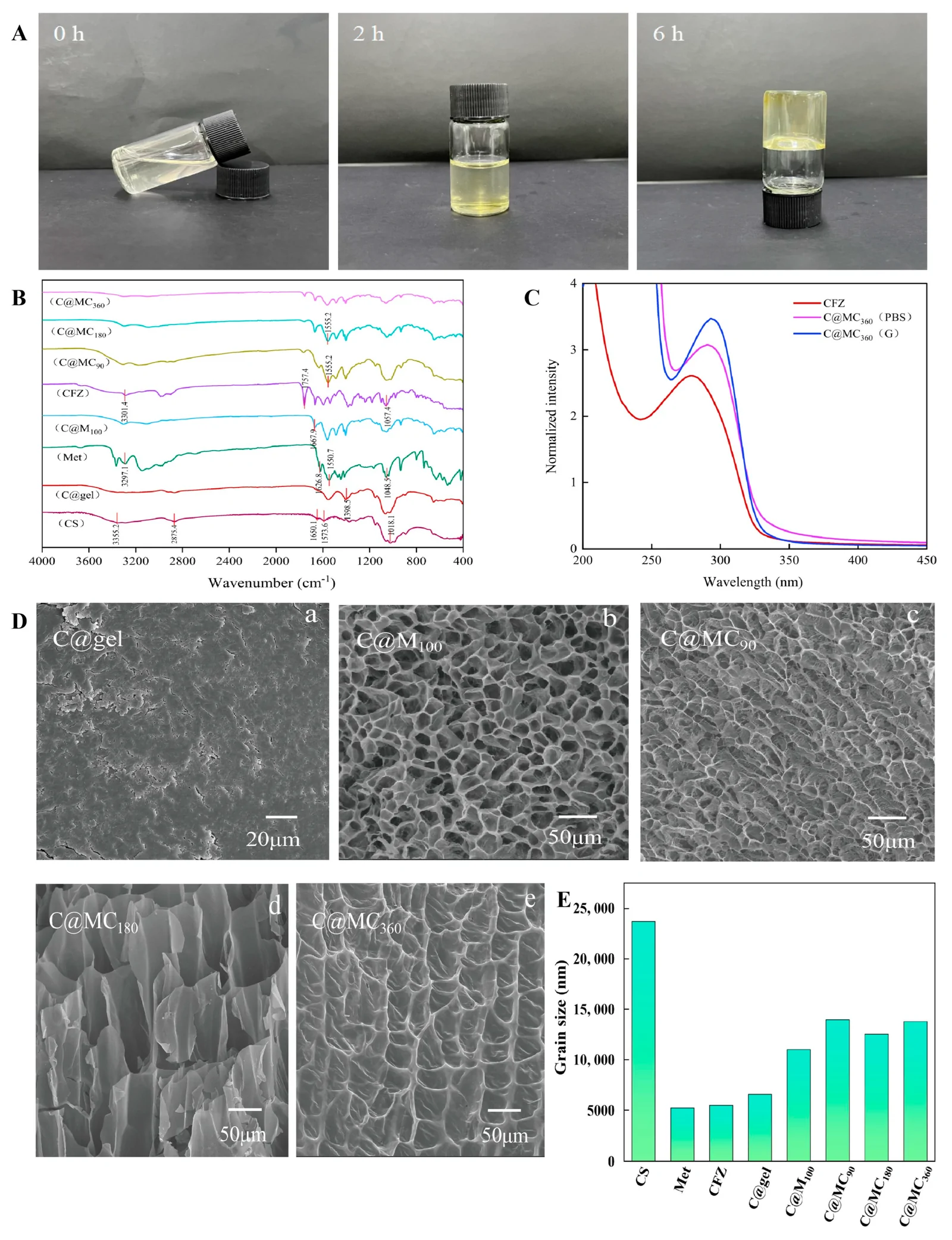
Characterization of hydrogels. (**A**) Time−lapse photographs showing sol−to−gel transition of C@MC_360_ hydrogel at 0 h (end of stirring, sol state), 2 h (partial supramolecular crosslinking), and 6 h (complete static gelation at room temperature without agitation). (**B**) FT−IR spectra of chitosan (CS), blank C@gel, metformin (Met), cefazolin sodium (CFZ), C@M_100_, C@MC_90_, C@MC_180_, and C@MC_360_. (**C**) UV−vis spectra of CFZ and its release from C@MC_360_ in PBS and glucose solution. (**D**) SEM images of hydrogels: C@gel (**a**), C@M_100_ (**b**), C@MC_90_ (**c**), C@MC_180_ (**d**), and C@MC_360_ (**e**). (**E**) Hydrodynamic aggregate size (nm) obtained from DLS measurement for CS, Met, CFZ, C@M_100_, C@MC_90_, C@MC_180_, and C@MC_360_. DLS data represent relative comparison of swollen hydrogel fragments rather than absolute particle size. Quantitative data are presented as mean ± SD; *n* = 3 independent replicates.

**Figure 2:**
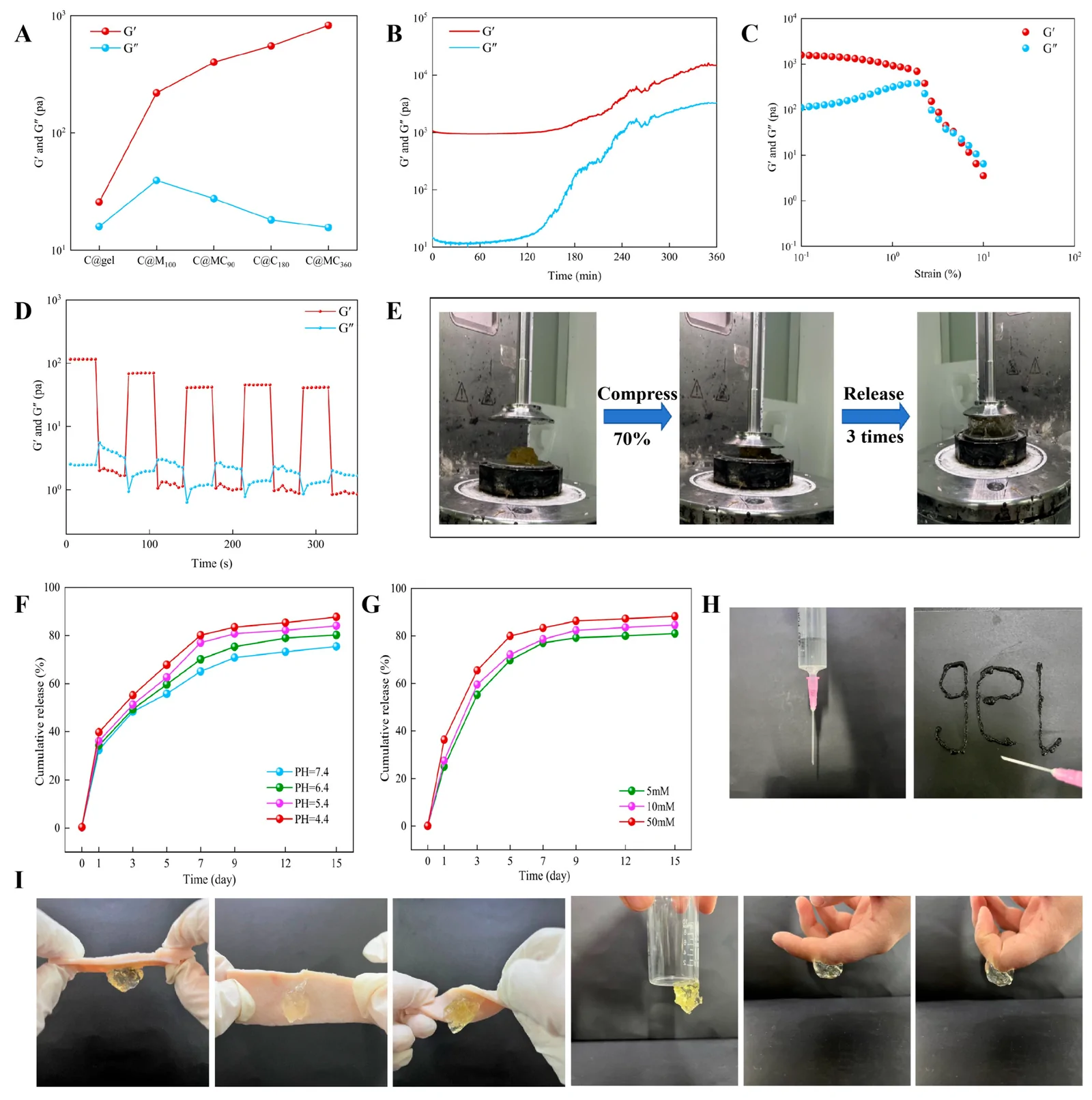
Rheological properties, injectability and tissue adhesion of C@MC hydrogels. (**A**) Storage modulus (G′) and loss modulus (G″) of C@gel, C@M_100_, C@MC_90_, C@MC_180_, and C@MC_360_ measured under 1% strain at 1 Hz. (**B**) Time sweep test of C@MC_360_ hydrogel during continuous oscillatory measurement over 6 h. (**C**) Strain amplitude sweep from 1% to 100% showing variations in G′ and G″ of C@MC_360_ hydrogel. (**D**) Step−strain tests to evaluate self−healing performance of C@MC_360_ hydrogel alternating between 1% and 100% strain for five cycles. (**E**) Three successive loading–unloading cycles at 70% strain for assessing compressive resilience of C@MC_360_. (**F**,**G**) Cumulative release of CFZ from C@MC_360_ hydrogel under different pH and glucose conditions. (**H**) Demonstration of injectability of C@MC_360_ through extrusion and writing. (**I**) Photographs displaying tissue adhesion of C@MC hydrogel on pig skin, finger joint and glass substrate. Quantitative rheology data are expressed as mean ± SD; *n* = 3 independent replicates.

**Figure 3:**
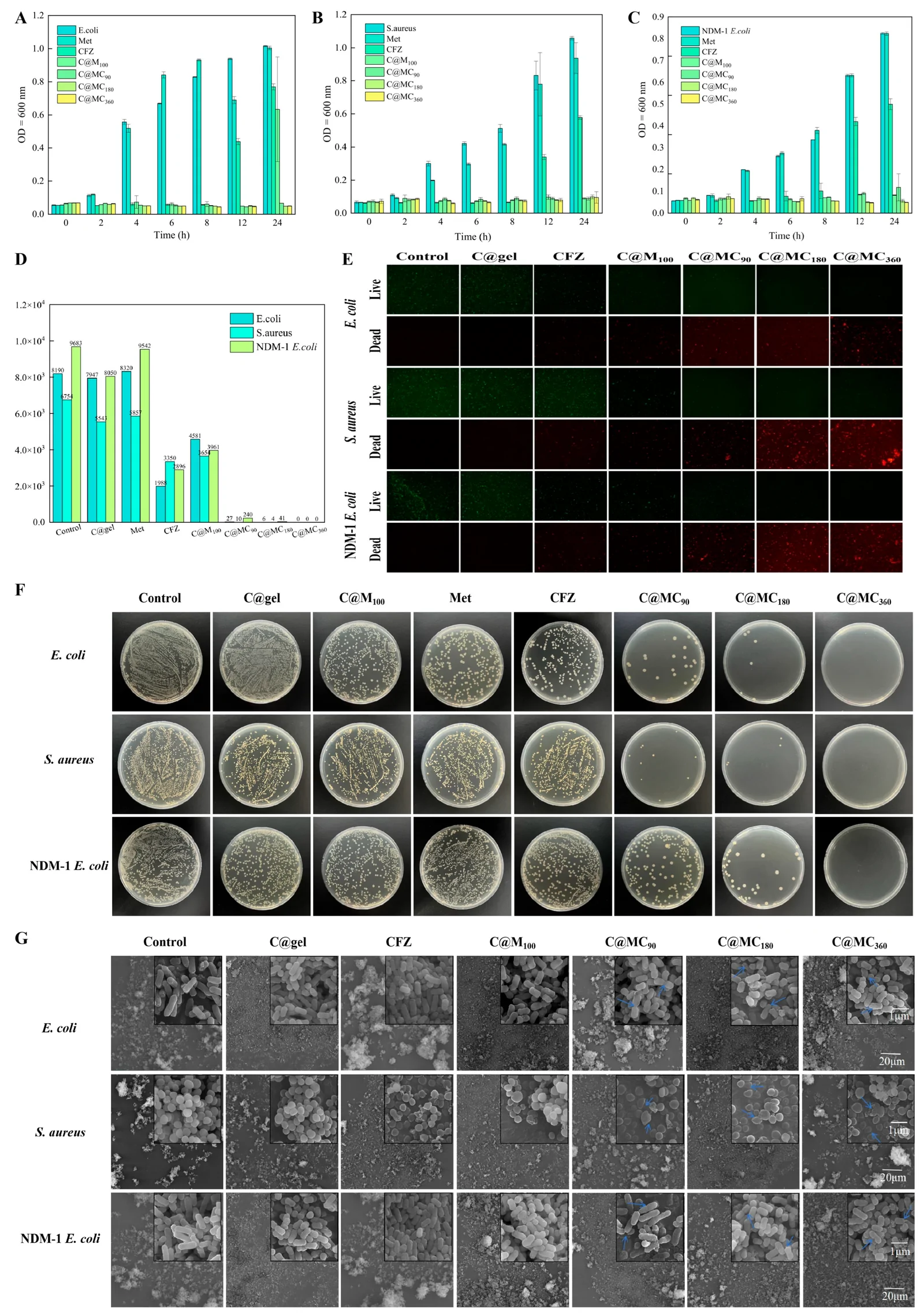
Antibacterial characterizations of C@MC hydrogels. (**A**–**C**) OD_600_ growth curves of *E. coli*, *S. aureus* and NDM-1 *E. coli*. (**D**) Colony-counting results of three bacteria subjected to PBS or hydrogels. (**E**) Live/dead fluorescence staining micrographs of bacteria cultured with different hydrogels: live bacteria (green), dead bacteria (red). (**F**) Photographs of agar plates cultivated with bacteria in PBS or hydrogels. (**G**) SEM images of three bacteria after treatment with PBS, C@gel, CFZ, C@M_100_, C@MC_90_, C@MC_180_, and C@MC_360_. Quantitative data (**A**–**D**) are presented as mean ± SD; *n* = 3 biological replicates per group.

**Figure 4:**
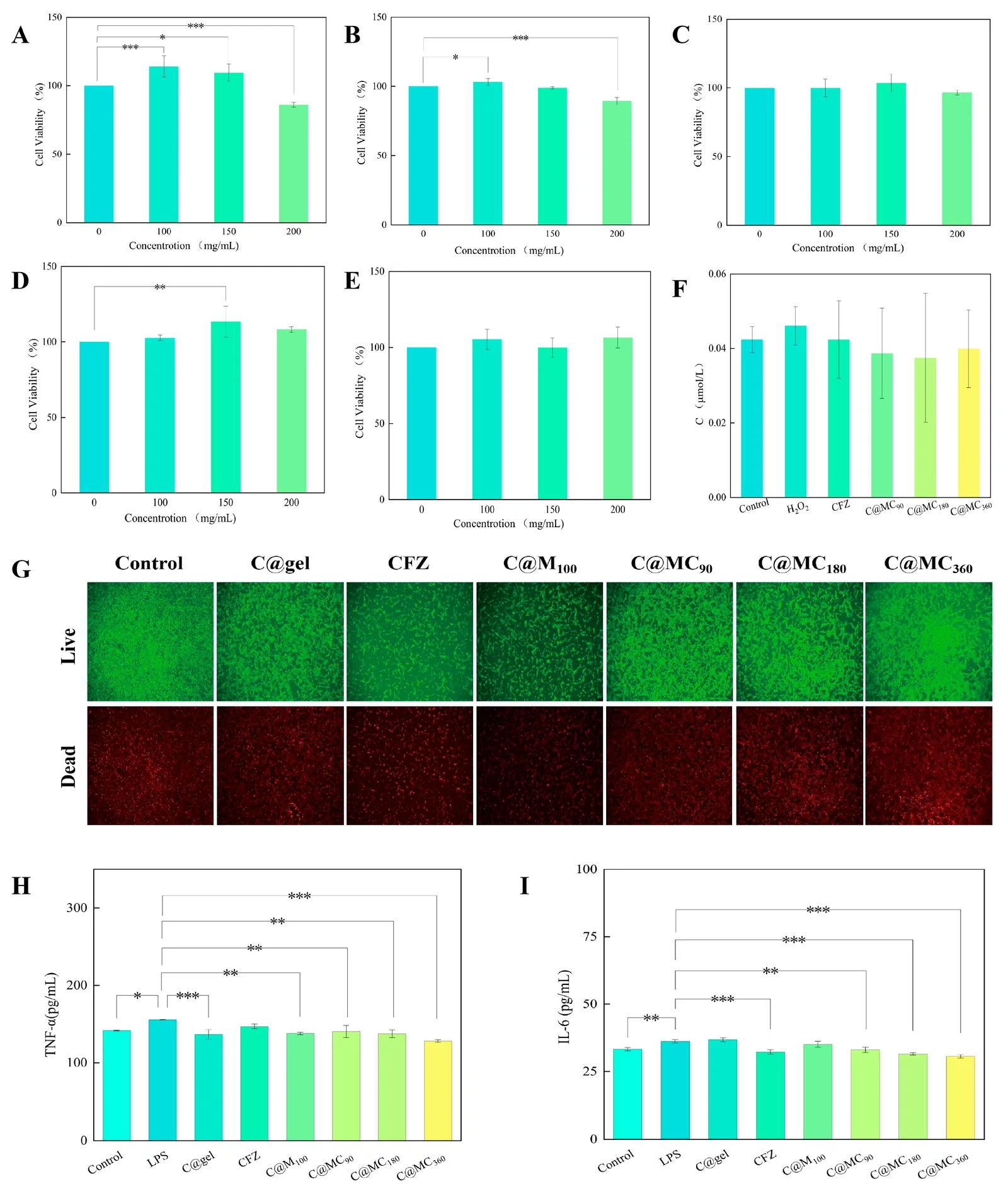
In vitro evaluation of hydrogels regarding biocompatibility and anti-inflammatory properties. (**A**–**E**) Cell viability of C_2_C_12_ cells after 24 h incubation with extracts from different hydrogels (C@gel, C@M_100,_ C@MC_90_, C@MC_180_, and C@MC_360_). (**F**) MDA levels in C_2_C_12_ cells following 24 h treatment with hydrogel extracts. (**G**) Live/dead fluorescent staining of C_2_C_12_ cells after hydrogel treatment for 24 h: live cells (green), dead cells (red). (**H**,**I**) ELISA quantification of inflammatory cytokines TNF-α (**H**) and IL-6 (**I**) in LPS-triggered RAW 264.7 macrophages preincubated with various hydrogel extracts. Values are mean ± SD (*n* = 3). Statistics: One-way ANOVA plus Tukey post hoc test. * *p* < 0.05, ** *p* < 0.01, *** *p* < 0.001.

**Figure 5:**
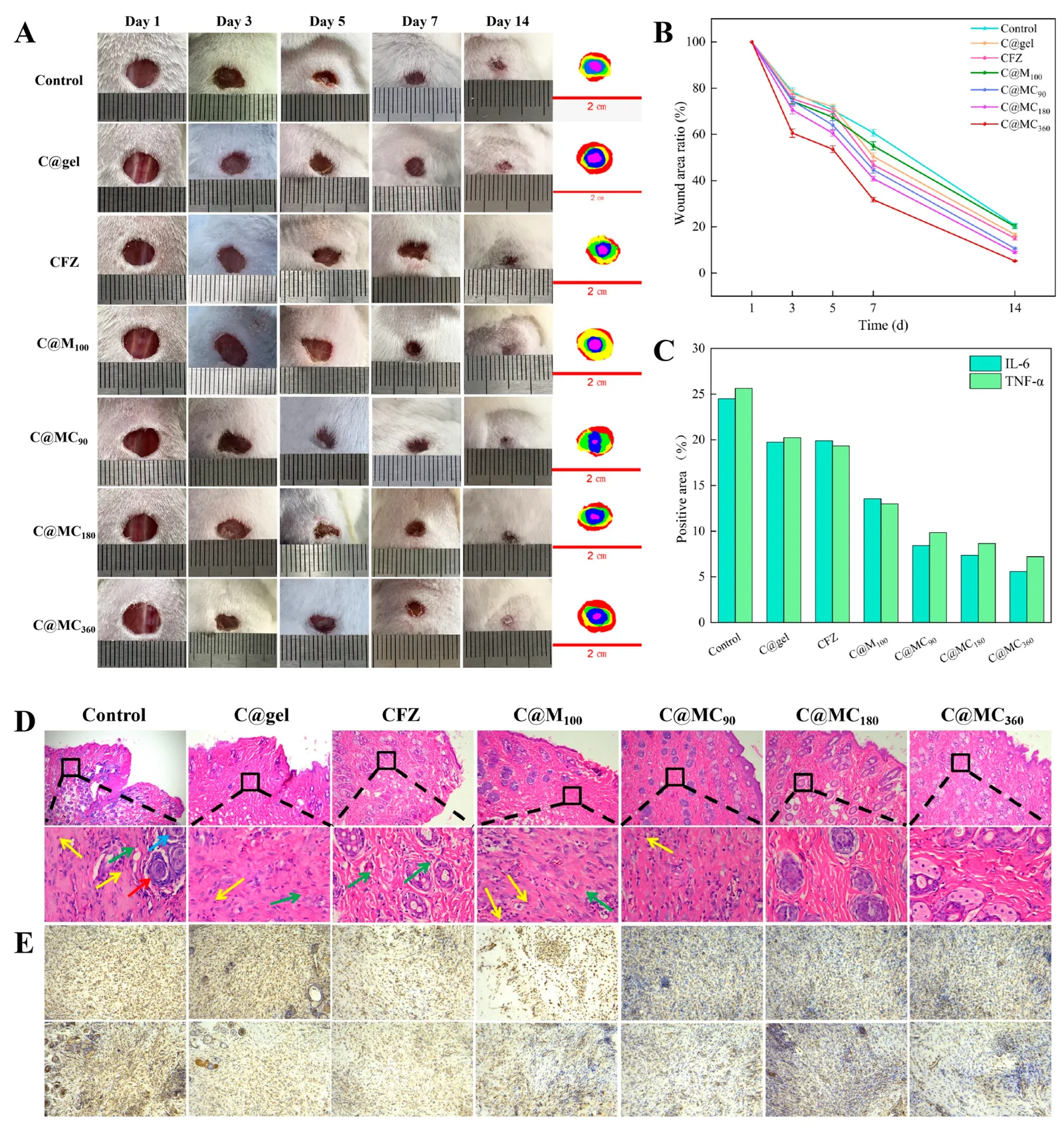
In vivo wound healing assessment of an *E. coli*-infected full-thickness skin defect mouse model. (**A**) Representative wound images and wound area heatmaps on days 0, 3, 5, 7, 14 post-injury. (**B**) Quantification analysis of residual wound area at predetermined time points. (**C**) ImageJ (version 1.54p) analysis of IL-6 and TNF-α immunohistochemical positive rates. (**D**) H&E staining of wound tissue at day 14 post-injury: yellow—neutrophils; green—lymphocytes; blue—plasma cells; red—macrophages. (**E**) Immunohistochemical staining of IL-6 and TNF-α in wound tissues. Quantitative data are expressed as mean ± SD; *n* = 8 mice per group.

**Figure 6:**
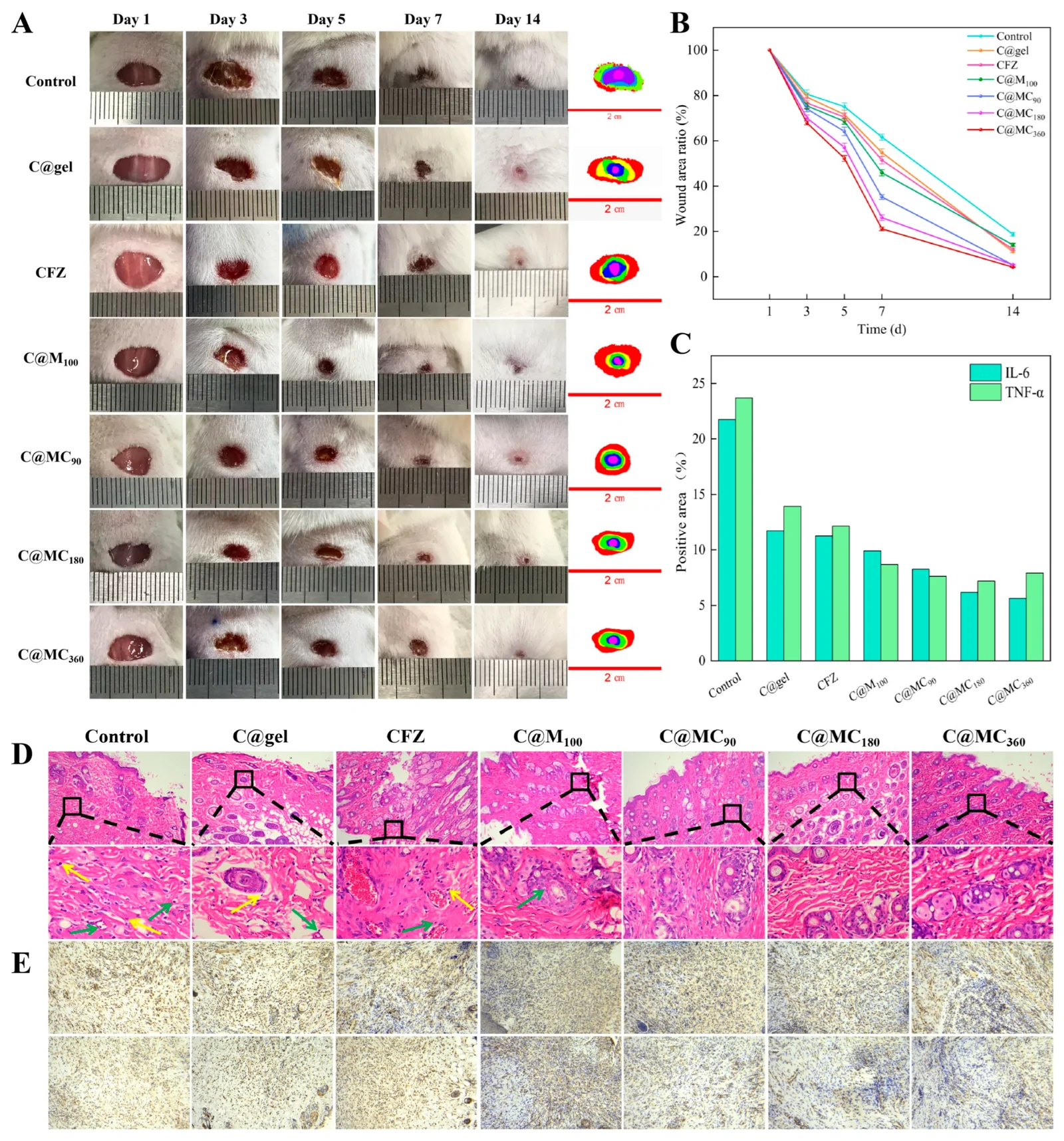
In vivo wound healing assessment of an NDM-1 *E. coli*-infected full-thickness skin defect mouse model. (**A**) Representative wound images and wound area heatmaps on days 0, 3, 5, 7, and 14 post-injury following infection with NDM-1 *E. coli.* (**B**) Wound closure percentages over time in different treatment groups. (**C**) Quantitative analysis of IL-6 and TNF-α immunohistochemical staining using ImageJ. (**D**) H&E histological staining of wound tissues harvested on day 14: yellow arrows indicate neutrophils; green arrows indicate lymphocytes. (**E**) Immunohistochemical staining visualization of IL-6 and TNF-α expression in wound tissue samples. Quantitative data are expressed as mean ± SD; *n* = 8 mice per group.

## Data Availability

The data that support the findings of this study are available from the corresponding author upon reasonable request.

## Supplementary Materials

The following supporting information can be downloaded at: https://www.mdpi.com/article/10.3390/gels12100876/s1, Figure S1: Scanning electron microscopy (SEM) images of various hydrogels at different magnifications. (a) C@M_10_ hydrogel; (b) C@M_50_ hydrogel; (c,d) C@MC_90_ hydrogel; (e,f) C@MC_180_ hydrogel; (g,h) C@MC_360_ hydrogel. C@M_10_ and C@M_50_ represent preliminary screened metformin-only hydrogels; C@M_100_ (shown in main text Figure 1D(b)) was finally selected as the optimized substrate for constructing dual-drug C@MC composite hydrogels; Figure S2: Zeta-potential distribution of different components and hydrogels. Zeta potential measurements were performed for CS, Met (metformin hydrochloride), CFZ (cefazolin sodium), blank C@gel, C@M_100_, C@MC_90_, C@MC_180_, C@MC_360_ to evaluate surface-charge characteristics of swollen hydrogel fragments. The near-neutral zeta-potential of optimized C@MC_360_ (0.614 mV) favors favorable cytocompatibility by avoiding excessive electrostatic interactions with negatively charged cell membranes, as described in Section 2.4 of the main manuscript; Figure S3: Standard curves and drug release behaviors of hydrogels under different conditions. (A,B) Standard calibration curves of Met (A) and CFZ (B). (C–F) Cumulative CFZ release profiles: C@MC_90_ under varied pH (C) and glucose concentrations (D); C@MC_180_ under varied pH (E) and glucose concentrations (F). (G–H) Cumulative Met release profiles of C@MC_360_ in PBS of different pH values (G) and media with different glucose concentrations (H). Sink conditions were maintained throughout all in-vitro release experiments. At predetermined time-points, 2 mL supernatant was withdrawn and replaced with an equal volume of pre-equilibrated fresh medium.
