# Supplementary material for: Dual-Drug Chitosan Hydrogel Loaded with Metformin and Cefazolin for Antibacterial, Anti-Inflammatory and Antioxidant Treatment of Drug-Resistant Bacteria-Infected Skin Wounds
Source: Gels. 2026 Sep 28;12(10):876. doi: 10.3390/gels12100876 (PMC13704149; doi:10.3390/gels12100876)
Supplement: Supplementary file 1 [file gels-12-00876-s001.zip › gels-4541832-supplementary.pdf]

Supplementary

Dual-Drug Chitosan Hydrogel Loaded with Metformin and Cefazolin for Antibacterial, Anti-Inflammatory and Antioxidant Treatment of Drug-Resistant Bacteria-Infected Skin Wounds

Yilin Zhang [1], Jie Cao [1], Yong Yan [1], Zhaoyang Li [2], Haiqiang Ren [1], Chenxiong Xu [1], Chengfang Qiao [2,*] and Wei Zhou [3]

[1] College of Modern Agriculture and Bioengineering, Shangluo University, Shangluo 726000, China; yilin_190@126.com (YL.Z.)
[2] College of Chemical Engineering and Modern Materials, Shangluo University, Shangluo 726000, China;
[3] Shaanxi Xiangju Pharmaceutical Group Company Limited, Shangluo 726000, China.
* Correspondence: xiaoqiaoqcf@126.com

1. Electron Microscopic Characterization of C@M and C@MC

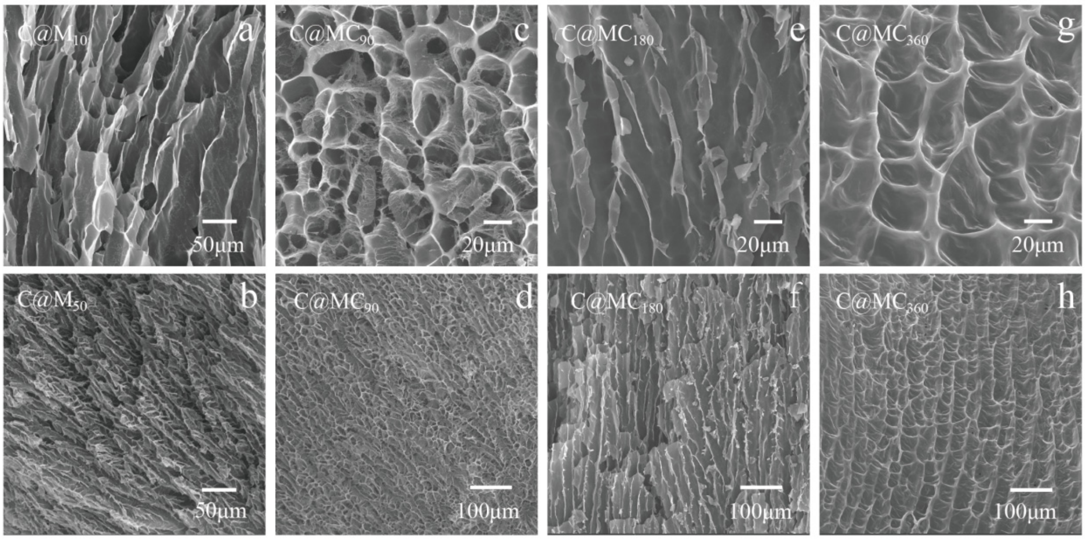

Figure S1. Scanning electron microscopy (SEM) images of various hydrogels at different magnifications.(a) $C@M_{10}$ hydrogel; (b) $C@M_{50}$ hydrogel;(c, d) $C@MC_{90}$ hydrogel; (e, f) $C@MC_{180}$ hydrogel; (g, h) $C@MC_{360}$ hydrogel. $C@M_{10}$ and $C@M_{50}$ represent preliminary screened metformin-only hydrogels; $C@M_{100}$ (shown in main text Figure 1D-b) was finally selected as the optimized substrate for constructing dual-drug C@MC composite hydrogels.

2. Zeta Potential of C@MC

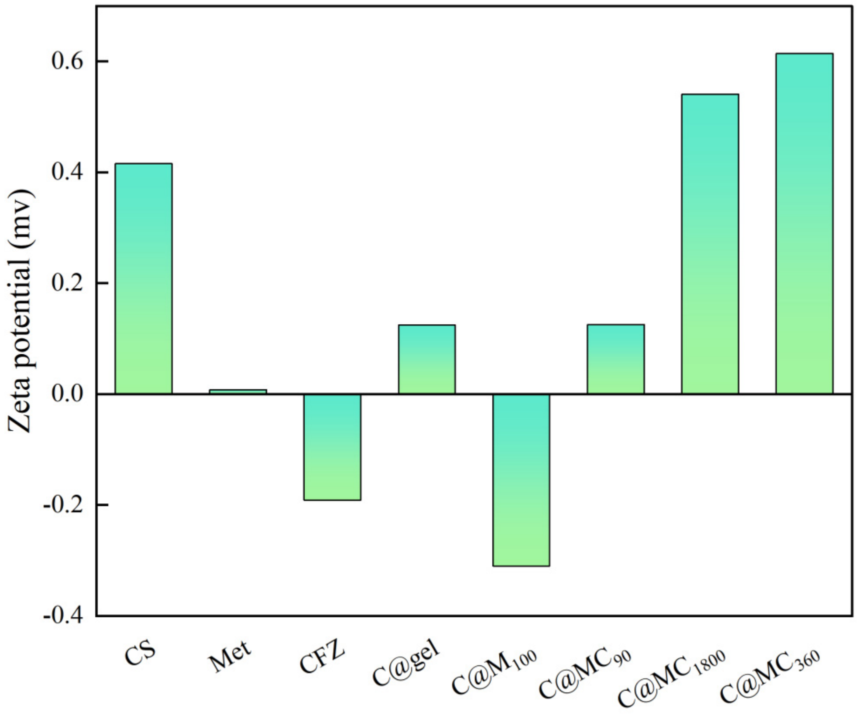

Figure S2. Zeta-potential distribution of different components and hydrogels. Zeta potential measurements were performed for CS, Met (metformin hydrochloride), CFZ (cefazolin sodium), blank C@gel, $C@M_{100}$, $C@MC_{90}$, $C@MC_{180}$, $C@MC_{360}$ to evaluate surface-charge characteristics of swollen hydrogel fragments. The near-neutral zeta-potential of optimized $C@MC_{360}$ (0.614 mV) favors favorable cytocompatibility by avoiding excessive electrostatic interactions with negatively charged cell membranes, as described in Section 2.4 of the main manuscript.

3. Study on Drug Release of C@MC

3.1. Preparation of Met and CFZ Standard Solution

Stock solutions (1 mg/mL) of Met and CFZ were prepared by dissolving powders in ultrapure water at room temperature. Series of working standard solutions were further diluted: Met: 5, 10, 15, 25, 50 μg/mL; CFZ: 50, 100, 150, 200, 250 μg/mL.

3.2. Determination of Maximum Absorption Wavelength

Ultrapure water served as blank reference. UV-vis scanning (200–800 nm) was performed. The maximum absorption wavelengths were confirmed: metformin hydrochloride λ_{max} = 232 nm, cefazolin sodium λ_{max} = 279 nm, consistent with Section 4.7 in the main manuscript.

3.3. Standard Curve Plotting

Absorbance values of serial standard solutions were detected at corresponding λmax according to the Lambert-Beer law. Calibration curves (Figure S3A,B) were plotted with concentration C (μg/mL) on the x-axis and absorbance A on the y-axis.

Met: $A = 0.08635C + 0.30172$, $R^2 = 0.99975$;

CFZ: $A = 0.01662C + 0.0501$, $R^2 = 0.99639$.

A = absorbance; C = concentration (μg/mL).

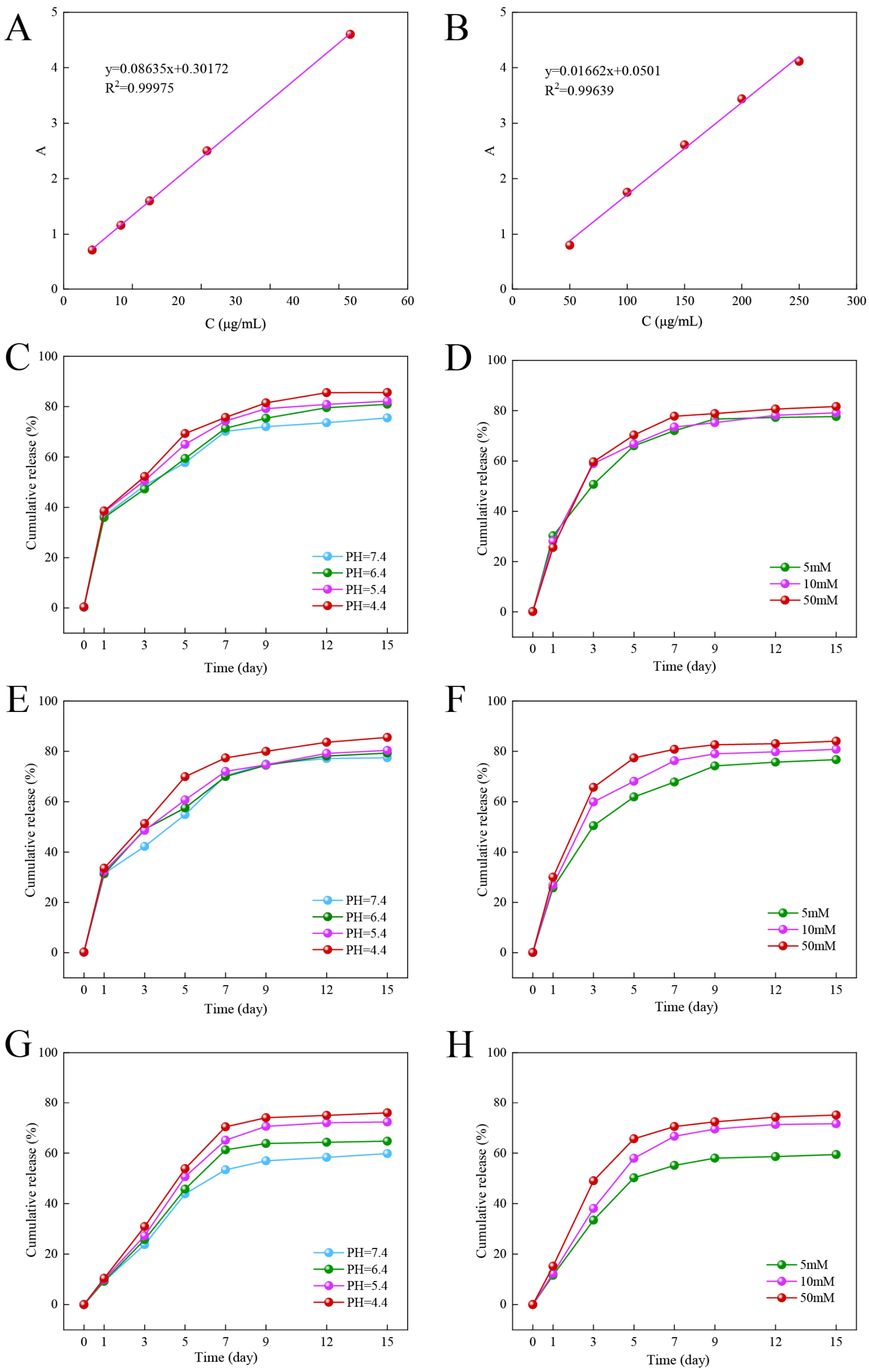

Figure S3. Standard curves and drug release behaviors of hydrogels under different conditions. (A, B) Standard calibration curves of Met (A) and CFZ (B). (C–F) Cumulative CFZ release profiles: C@MC90 under varied pH (C) and glucose concentrations (D); C@MC180 under varied pH (E) and glucose concentrations (F). (G-H) Cumulative Met release profiles of C@MC360 in PBS of different pH values (G) and media with different glucose concentrations (H). Sink conditions were maintained throughout all in-vitro release experiments. At predetermined time-points, 2 mL supernatant was withdrawn and replaced with an equal volume of pre-equilibrated fresh medium.
